# Estimating the Effective Population Size from Temporal Allele Frequency Changes in Experimental Evolution

**DOI:** 10.1534/genetics.116.191197

**Published:** 2016-08-19

**Authors:** Ágnes Jónás, Thomas Taus, Carolin Kosiol, Christian Schlötterer, Andreas Futschik

**Affiliations:** *Vienna Graduate School of Population Genetics, 1210 Vienna, Austria; †Institut für Populationsgenetik, Vetmeduni Vienna, 1210 Vienna, Austria; ‡Department of Applied Statistics, Johannes Kepler Universität Linz, 4040 Linz, Austria

**Keywords:** effective population size, genetic drift, Pool-seq, experimental evolution

## Abstract

The effective population size ($N_{e}$) is a major factor determining allele frequency changes in natural and experimental populations. Temporal methods provide a powerful and simple approach to estimate short-term $N_{e}.$ They use allele frequency shifts between temporal samples to calculate the standardized variance, which is directly related to $N_{e}.$ Here we focus on experimental evolution studies that often rely on repeated sequencing of samples in pools (Pool-seq). Pool-seq is cost-effective and often outperforms individual-based sequencing in estimating allele frequencies, but it is associated with atypical sampling properties: Additional to sampling individuals, sequencing DNA in pools leads to a second round of sampling, which increases the variance of allele frequency estimates. We propose a new estimator of $N_{e},$ which relies on allele frequency changes in temporal data and corrects for the variance in both sampling steps. In simulations, we obtain accurate $N_{e}$ estimates, as long as the drift variance is not too small compared to the sampling and sequencing variance. In addition to genome-wide $N_{e}$ estimates, we extend our method using a recursive partitioning approach to estimate $N_{e}$ locally along the chromosome. Since the type I error is controlled, our method permits the identification of genomic regions that differ significantly in their $N_{e}$ estimates. We present an application to Pool-seq data from experimental evolution with *Drosophila* and provide recommendations for whole-genome data. The estimator is computationally efficient and available as an R package at https://github.com/ThomasTaus/Nest.

DURING experimental evolution studies, populations are maintained under specific laboratory conditions (Kawecki *et al.* 2012; Long *et al.* 2015; Schlötterer *et al.* 2015). In sexually reproducing organisms, the census population size is typically kept fixed at fairly low numbers, rarely exceeding 2000 individuals. With such small population sizes, genetic drift causes stochastic fluctuations in allele frequencies. Under neutrality, the level of random frequency changes is determined by the effective population size ($N_{e}$) (Wright 1931). Furthermore, the efficacy of selection is influenced by $N_{e}.$ For weakly selected alleles, the probability of fixation is directly proportional to the product of $N_{e}$ and the intensity of selection (Fisher 1930; Kimura 1964). As changes in allele frequency are greatly affected by the population size, it is fundamental to estimate $N_{e}$ accurately to understand molecular variation in experimental evolution studies.

Krimbas and Tsakas (1971) estimated $N_{e}$ using the standardized variance of allele frequency (*F*, see also Falconer and Mackay 1996) from longitudinal samples in natural populations of olive flies. As *F* was calculated from these samples, they accounted for the sampling variance that also contributed to the true allele frequency variance. This approach was further improved and used by several authors (Nei and Tajima 1981; Pollak 1983; Waples 1989; Jorde and Ryman 2007).

With the widespread availability of powerful computers, also maximum-likelihood-based methods became popular (Williamson and Slatkin 1999; Anderson *et al.* 2000; Wang 2001; Hui and Burt 2015) in addition to the moment-based approaches discussed above. Although these methods show less bias than the moment-based approaches (Wang 2001), they are still computationally demanding, in particular for the large numbers of markers typically obtained with novel sequencing technologies (Foll *et al.* 2015).

Estimating $N_{e}$ with temporal methods requires samples collected at least at two time points. Alternative methods that use only a single time point are based on linkage disequilibrium (LD) (Hill 1981; Waples and Do 2008, 2010; Waples and England 2011), heterozygote excess (Pudovkin *et al.* 1996), molecular coancestry (Nomura 2008), sibship frequencies (Wang 2009, 2013), or combinations of summary statistics using approximate Bayesian computation (Tallmon *et al.* 2008). LD-based methods are widespread but require haplotype or unphased diploid genotype information, which limits their applicability.

Although the cost for sequencing has dropped considerably, the separate sequencing of thousands of individuals in replicate populations in experimental evolution studies is still out of reach. Sequencing samples in pools (Pool-seq) can provide a cost-effective alternative (Schlötterer *et al.* 2014). Pool-seq has also been shown to outperform individual-based sequencing in estimating allele frequencies and inferring population genetic parameters under several conditions (Futschik and Schlötterer 2010; Zhu *et al.* 2012; Gautier *et al.* 2013). For these reasons, Pool-seq has become the basis of many experimental evolution “evolve and resequence” (E&R) studies (Turner *et al.* 2011; Schlötterer *et al.* 2015). Following the emergence of E&R, many population genetic estimators have been adjusted to handle the properties of Pool-seq data (Futschik and Schlötterer 2010; Kofler *et al.* 2011a,b; Kolaczkowski *et al.* 2011; Boitard *et al.* 2013; Ferretti *et al.* 2013). To the best of our knowledge, no $N_{e}$ estimators have been developed so far that properly deal with Pool-seq data.

In this article, we present a novel temporal method to estimate $N_{e}$ from pooled samples. We show that previously proposed estimators can lead to substantial bias, as they neglect the variance component due to pooled sequencing. We introduce a new model accounting for the two-step sampling process associated with Pool-seq data. In the first sampling step individuals are drawn from the population to create pooled DNA samples. In the second step, pooled sequencing is modeled as binomial sampling of reads out of the DNA pool. We show on simulated data that our method outperforms standard temporal $N_{e}$ estimators. For real data, we also suggest to use a segmentation algorithm, to partition the genome-wide sequence data into stretches of DNA with significantly different $N_{e}$ estimates. Finally, we present an application to a genome-wide experimental evolution data set from *Drosophila melanogaster* (Franssen *et al.* 2015).

## Materials and Methods

### Sampling schemes

Nei and Tajima (1981) investigated the sampling properties of temporal $N_{e}$ estimators and proposed two different sampling schemes. Under the first scheme (plan I), individuals are either sampled after reproduction or returned to the population after their genotypes have been examined. In contrast, under the second scheme (plan II) sampling takes place before reproduction and the sampled individuals are permanently removed from the population and their genotypes do not contribute to the next generation. By assuming different sampling distributions, they derived separate $N_{e}$ estimators under sampling plans I and II.

Waples (1989) unified the calculations under the two plans by assuming binomial sampling out of an infinitely large parental gamete pool for both sampling schemes. He concluded that the measure of variance under the two sampling plans differs only in a covariance term. For plan I, there is a positive correlation between allele frequencies sampled *t* generations apart because they are both derived from the same population at generation 0. In contrast, for plan II, the initial sample and individuals contributing to the next generation can be considered as independent binomial samples; thus sample frequencies at generations 0 and *t* are uncorrelated.

**Figure 1:**
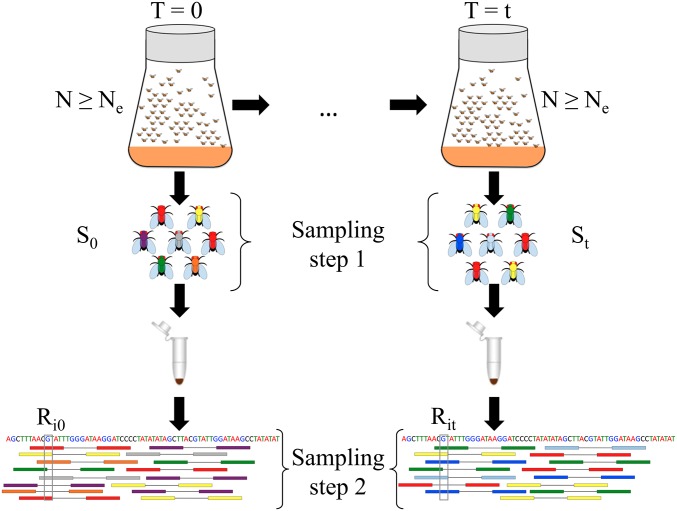
Two-step sampling in experimental evolution with *Drosophila*. In E&R studies, populations are propagated at a census size *N* defined by the experimenter, which is in general larger than the effective population size $N_{e}.$ Using temporal methods, $N_{e}$ can be estimated from the variance in allele frequency between samples taken *t* generations apart. To get an accurate representation of allele frequencies in population genetic studies, a large number of individuals $S_{j}$ (j∈{0,t}) are sampled and pooled. Sampling can take place according to sampling plan I or II based on the mode of reproduction. Pooled samples are then subjected to high-throughput sequencing. Sequenced reads are subsequently aligned to the reference genome (shown at the bottom). We represent pool sequencing by an additional sampling step (called sampling step 2). We correct for both sampling steps when estimating $N_{e}$ in pooled samples. Additionally, we take into account variable coverage levels across the genome (coverage $R_{ij}$ for site *i* at T=j, j∈{0,t}) when correcting for the variance coming from sequencing.

For a typical E&R study, outbred experimental populations are created by mixing a large number of inbred lines (*e.g.*, Turner and Miller 2012; Huang *et al.* 2014; Franssen *et al.* 2015). The populations are then propagated under the desired experimental conditions while keeping the census size of the population controlled through time (Figure 1). However, the experimenter has no direct influence on the effective population size, which is in general lower than the census size. In E&R studies with *Drosophila*, the census size rarely exceeds some hundreds of individuals, and sampling usually takes place after reproduction according to plan I. For organisms maintained at larger sizes, such as yeast, the sample for genetic analysis is not returned to the population (Burke *et al.* 2014). Plan II applies to such cases.

In E&R studies, sampled individuals are often pooled together for DNA extraction (Schlötterer *et al.* 2014). The size of the pool can be as large as the whole population. Depending on the experimental design, it is also possible that only a fraction of the population is sequenced, for instance, only females (Tobler *et al.* 2014; Franssen *et al.* 2015). Pooled individuals are used to create DNA libraries, which are, in turn, subjected to high-throughput sequencing.

We consider two separate sampling steps when estimating $N_{e}$ from Pool-seq samples (Figure 1). In the first step, we model the sampling of individuals out of the population. This can take place according to either plan I or plan II. In the second step, we model the sequencing of a DNA pool by drawing reads at random with replacement from the first-step sample. The allele frequency variance inferred from the sample is corrected for the additional variance coming from the two-step sampling and used for estimating $N_{e}.$

### Notation

We assume that the experimental population is propagated at a constant census size *N* and that $N\geq N_{e}.$ We use genome-wide single-nucleotide polymorphism (SNP) data sampled *t* generations apart to estimate $N_{e}$ (Figure 1) and denote the estimated effective population size by ${\hat{N}}_{e}.$ Multiallelic sites in populations with low mutation rates, such as *Drosophila*, exist but are rare and likely to be sequencing errors (Burke *et al.* 2010). Therefore we consider only biallelic SNPs at *n* polymorphic sites. At each site *i* (i=1…n) the true population allele frequency is denoted by $p_{ij}$ at time T=j, where j∈{0,t}. To obtain allele frequency estimates for an unknown $p_{ij},$ the population is subjected to sampling. We consider two sampling steps (Figure 1). At T=j, we first sample $S_{j}$ individuals out of the population to create a pooled DNA library for sequencing. Note that the number of sampled individuals is constant over the *n* sites, and therefore the index *i* is omitted here. Sampling individuals can take place according to either plan I or plan II, as described above (also shown in Supplemental Material, Figure S1). As the second sampling step, we model Pool-seq by drawing $R_{ij}$ reads out of the pooled DNA sample at each site *i* (i=1…n). This allows for variation in sequence coverage. Below we derive the variance in allele frequency for a given site. To keep notation simple, we omit again the index *i* and denote the unknown sample allele frequency among the $S_{0}$ individuals at the first sampling time point (T=0) by *x* and the subsequent allele frequency estimate obtained via pool sequencing from $R_{0}$ reads by $\hat{x}.$ Similarly, at some T=t, the respective frequencies are denoted by *y* and $\hat{y}.$ Note that under pool sequencing only $\hat{x}$ and $\hat{y}$ are observed.

### Estimating $N_{e}$ from temporal allele frequency changes

Under neutral Wright–Fisher evolution the variance in allele frequency ($\sigma_{p}^{2}$) generated by drift after *t* generations at a single locus in a diploid population is well described by the expression

$$\sigma_{p}^{2}=p\left(1-p\right)\left[1-{\left(1-\frac{1}{2N_{e}}\right)}^{t}\right],$$ (1)

where *p* is the starting allele frequency (Falconer and Mackay 1996). Wright (1931) denoted the standardized variance by $F=\sigma_{p}^{2}/p\left(1-p\right),$ which leads to a convenient closed-form expression for $N_{e}.$ Furthermore, if $N_{e}$ is large enough, $F\approx 1-e^{-t/2N_{e}}$ and $N_{e}$ can be calculated as

$$N_{e}\approx \frac{-t}{2\;\ln\left(1-F\right)}.$$ (2)

The relation between $N_{e}$ and allele frequency changes described in Equation 1 was first used by Krimbas and Tsakas (1971) in natural populations of olive flies. They estimated the variance using

$$F=F_{a}:=\frac{1}{a}\sum_{k=1}^{a}\frac{{\left(x_{k}-y_{k}\right)}^{2}}{x_{k}\left(1-x_{k}\right)}\;,$$ (3)

where $x_{k}$ and $y_{k}$ (k=1,…,a) are the observed allele frequencies in the samples collected *t* generations apart and *a* is the number of alleles at a specific locus. To eliminate the contribution of sampling errors to the variance, the total variance $F_{a}$ was corrected for the random sampling noise by simply subtracting the corresponding variance. This approach was further investigated and developed by a number of authors (Pamilo and Varvio-Aho 1980; Nei and Tajima 1981; Pollak 1983; Waples 1989).

Possible sources of bias in $N_{e}$ estimators were later investigated by Jorde and Ryman (2007). The authors pointed out that the expectation over *F* is typically approximated by taking the expected values separately for the numerator and the denominator (Turner *et al.* 2001). They suggested a different weighting scheme of alleles leading to an alternative less-biased estimator to measure temporal frequency change.

### Correction for two-step sampling

We consider a random-mating population with discrete generations. Neutral evolution is assumed with no selection, migration, and mutation. Samples are drawn from the population at generations T=0 and *t*. Throughout the derivation we consider diploid populations, and therefore a sample of $S_{j}$ individuals leads to $2S_{j}$ sequences at times T=j∈{0,t}. Sampling is assumed to be binomial with parameters $2S_{j}$ and $p_{j}$ (Waples 1989). In the second sampling step at time T=j, sequencing a random pool $R_{j}$ of reads is also modeled as binomial sampling.

In analogy to Jorde and Ryman (2007), we use the following expression as our measure of the temporal change in allele frequency for biallelic sites,

$$F_{c}=\frac{{\left(\hat{x}-\hat{y}\right)}^{2}}{\hat{z}-\hat{x}\hat{y}},$$ (4)

where $\hat{z}=\left(\hat{x}+\hat{y}\right)/2.$

The expectation of $F_{c}$ for a single biallelic locus is approximated by

$$E\left(F_{c}\right)\approx \frac{E{\left(\hat{x}-\hat{y}\right)}^{2}}{E\left(\hat{z}-\hat{x}\hat{y}\right)}=\frac{\mathrm{Var}\left(\hat{x}\right)+\mathrm{Var}\left(\hat{y}\right)-2\mathrm{Cov}\left(\hat{x},\hat{y}\right)}{E\left(\hat{z}-\hat{x}\hat{y}\right)}.$$ (5)

For both plans, we derive expressions for the numerator and denominator in Equation 5 separately under the two-step sampling procedure, described above. Here we summarize our main conclusions; details on the derivation are provided in File S1. With $C_{j}:=1/2S_{j}+1/R_{j}-1/2S_{j}R_{j}$ for j∈{0,t}, and *p* denoting the true population allele frequency in the gamete pool at generation 0, we obtain

$$\mathrm{Var}\left(\hat{x}\right)=p\left(1-p\right)C_{0},$$ (6)

and

$$\mathrm{Var}\left(\hat{y}\right)=p\left(1-p\right)\left[1-\left(1-C_{t}\right){\left(1-\frac{1}{2N_{e}}\right)}^{t}\right].$$ (7)

Note that Equations 6 and 7 differ only in the correction term $C_{j}$ from that in Waples (1989).

Waples (1989) previously showed that the denominator in Equation 5 reduces to

$$E\left(\hat{z}-\hat{x}\hat{y}\right)=p\left(1-p\right)-\mathrm{Cov}\left(\hat{x},\hat{y}\right).$$ (8)

For plan II, $\mathrm{Cov}\left(\hat{x},\hat{y}\right)=0$ (Waples 1989), and $F_{c}$ corrected for the noise coming from the two-step sampling for a single locus is given by

$${F^{\prime }}_{c}=\frac{F_{c}-C_{0}-C_{t}}{1-C_{t}}.$$ (9)

For plan I, on the other hand, the sample allele frequency at generation 0 is positively correlated to the sample allele frequency at *t* because both are derived from the same population at generation 0. This requires us to calculate the sample covariance $\mathrm{Cov}\left(\hat{x},\hat{y}\right)$ in Equation 5. It turns out (see File S1 for details) that the covariance of $\hat{x}$ and $\hat{y}$ is equal to

$$\mathrm{Cov}\left(\hat{x},\hat{y}\right)=\frac{p\left(1-p\right)}{2N},$$ (10)

where *N* is the census size of the population at generation 0. Equation 10 is in agreement with the corresponding term of the standard methods (Waples 1989). Substituting the inferred covariance into Equation 5 leads to the following corrected variance estimate, ${F^{\prime }}_{c}$ for plan I

$${F^{\prime }}_{c}=\frac{F_{c}\left(1-1/2N\right)-C_{0}-C_{t}+1/N}{1-C_{t}}\;.$$ (11)

We provide the corresponding formulas of ${F^{\prime }}_{c}$ in haploid populations in File S1.

With Pool-seq data, randomness in sequencing and local structures in the genome can lead to different coverage across marker sites, which we denote by $R_{ij}$ for site *i* (i=1,…n) and time *j* (j∈{0,t}). In the genome-wide data set, we calculate ${F^{\prime }}_{c}$ across *n* SNPs by summing over all loci in the numerator and denominator separately before carrying out the division in Equation 9, leading to the following weighting scheme for plan II:

$$\bar{{F^{\prime }}_{c}}=\frac{\sum_{i=1}^{n}{\left({\hat{x}}_{i}-{\hat{y}}_{i}\right)}^{2}-\left({\hat{z}}_{i}-{\hat{x}}_{i}{\hat{y}}_{i}\right)\left(C_{i0}+C_{it}\right)}{\sum_{i=1}^{n}\left({\hat{z}}_{i}-{\hat{x}}_{i}{\hat{y}}_{i}\right)\left(1-C_{it}\right)}.$$ (12)

Similarly, $\bar{{F^{\prime }}_{c}}$ can be calculated for plan I using Equations 4 and 11. Analogous to the single-locus case, our proposed estimators ${\hat{N}}_{e}\left(P\right)$ for a diploid population are obtained by plugging $\bar{{F^{\prime }}_{c}}$ into Equation 2.

Long time series have recently become available for some E&R experiments (Barrick *et al.* 2009; Burke *et al.* 2010, 2014). Standard $N_{e}$ estimators (Krimbas and Tsakas 1971; Nei and Tajima 1981; Waples 1989) assume a small number of generations (t) and approximate $N_{e}$ using $2N_{e}\approx t/F.$ If, however, $t/N_{e}$ is larger, using this approximation can lead to severe bias (Figure S2). To avoid such a bias, we use Equation 2 to estimate $N_{e}.$

### Simulations

We evaluate the performance of our estimator on data simulated under the neutral Wright–Fisher model. With a given population size of $2N_{e},$ we simulate the frequency trajectory of *n* independent SNPs. As we focus on biallelic SNPs, we assume two possible nucleotides (alleles) to be present in the population with given frequencies at the start. To create a new generation, nucleotides are drawn independently at random with a probability given by their respective allele frequencies in the old generation. The population is propagated at a constant size of $2N_{e}$ for *t* nonoverlapping generations. The effective population size is then estimated from allele frequencies inferred from Pool-seq samples taken from the population at the start and after *t* generations. The sampling of individuals to create the pooled DNA library is simulated by using sampling without replacement. To model the uneven coverage of genome-wide sequence data, we simulate a random coverage for each site, using a Poisson distribution with parameter equal to the given mean coverage. For every position, reads are then generated by binomial sampling from the library with sample size equal to the local coverage.

We assess the performance of our estimator for various combinations of $N_{e},$ pool size, coverage, number of SNPs, and distribution of starting allele frequencies. Additional to these parameters, we also test how the ratio between census and effective population size ($r=N/N_{e}$) affects the accuracy of the proposed estimator. For this purpose, we increment the population size to a desired level of *N* in each generation while keeping the allele frequencies unchanged to avoid introducing additional sampling variance. We simulated each scenario 100 times.

Linkage disequilibrium between loci can reduce the number of independent SNPs, thereby increasing the variance of the estimate. The impact of dependence between SNPs is investigated based on 10 replicated whole-genome forward simulations with recombination, using the software tool MimicrEE (Kofler and Schlötterer 2014). As a starting population for the forward simulations, we sampled 2000 haploid genomes out of 8000 genomes simulated with fastsimcoal v.1.1.2 (Excoffier and Foll 2011; Bastide *et al.* 2013). The parameters used to generate the genomes mimic a wild population of *D. melanogaster* from Vienna (Fiston-Lavier *et al.* 2010; Bastide *et al.* 2013; Kofler and Schlötterer 2014). Allele counts are subjected to binomial sampling to mimic Pool-seq with a given sequence coverage. $N_{e}$ is estimated in nonoverlapping windows, each containing a fixed number of SNPs.

### Estimating $N_{e}$ on simulated data

We denote our estimator corrected for the additional sampling step, *i.e.*, pooling, by $N_{e}\left(P\right).$ We compare $N_{e}\left(P\right)$ to the standard estimators $N_{e}\left(W\right)$ and $N_{e}\left(JR\right)$ proposed by Waples (1989) and Jorde and Ryman (2007) that correct only for a single sampling step.

We illustrate experimental sampling procedures by considering two major scenarios: (i) The full population is sequenced as one large pool and (ii) only a subset of the population is used to create pooled samples. Under scenario (i) we simulate only a single binomial sampling step to represent sampling reads out of the DNA pool. The pool size is set to be equal to the census size of the population ($S_{j}=N$), and the number of sampled reads ($R_{ij}$) represents the per-site coverage. For estimators that correct only for a single sampling step, we use the coverage ($R_{ij}$) as the sample size. For scenario (ii), the sampled individuals ($S_{j}$) and the read number ($R_{ij}$) represent the pool size and coverage for $N_{e}\left(P\right).$ The coverage ($R_{ij}$) is taken as the sample size for the $N_{e}\left(W\right)$ and $N_{e}\left(JR\right)$ estimators, as these methods consider only one sampling step.

### Change point inference for genome-wide estimates

The effect of genetic drift on the variance in allele frequency specified in Equation 1 holds only under the assumptions of Wright–Fisher neutral evolution. Deviations from the Wright–Fisher model, such as the presence of selection or demography, may cause systematically different changes in allele frequency, affecting the variance and causing locally variable patterns in genetic diversity. Furthermore, the effect of selection on one site of the genome may cause changes in the behavior of variants at nearby sites (Maynard Smith and Haigh 1974; Barton 2000; Comeron *et al.* 2008). As a result, the estimates of $N_{e}$ at different locations of the genome may deviate from the true number of breeding individuals in the population (Kimura and Crow 1963; Charlesworth 2009). For example, regions under background selection are associated with reduced ${\hat{N}}_{e}$ values that extend to linked sites due to the Hill–Robertson effect (Charlesworth 1996, 2012a; Comeron *et al.* 2008). Similarly, selectively favorable alleles can also drag nearby neutral sites to high frequency (Maynard Smith and Haigh 1974), causing a local reduction in the estimated $N_{e}$ (Liu and Mittler 2008). Such an event is also known as a selective sweep (Berry *et al.* 1991). On the other hand, we expect the opposite pattern, *i.e.*, a local elevation of ${\hat{N}}_{e}$ for types of selection such as balancing selection that maintain variation in the genome (Baysal *et al.* 2007; Charlesworth 2009).

To detect such patterns in ${\hat{N}}_{e},$ we apply a segmentation algorithm to partition the genome into locally homogeneous ${\hat{N}}_{e}$ stretches. We use a method related to an approach suggested by Futschik *et al.* (2014) for partitioning DNA sequences with respect to GC content. It is based on a statistical multiscale criterion and provides statistical error control, in the sense that the estimated number of windows will not exceed the true one except for a small error probability *α* to be specified by the user. With our $N_{e}$ estimates, we use a criterion proposed by Frick *et al.* (2014) for normally distributed responses. It is implemented as part of the R package *stepR* (Frick *et al.* 2014). By using simulations with selection we also illustrate that this method is able to capture the signal of locally variable ${\hat{N}}_{e}$ along the chromosome.

### Data availability

We estimated *N*_e_ in an E&R study with *D. melaongaster*, published in Orozco-terWengel *et al.* (2012) and Franssen *et al.* (2015). Pool-seq read libraries from these studies are available at the European Sequence Read Archive at http://www.ebi.ac.uk/ena/ under accession nos. ERP001290 and ERS460611–ERS460613.

## Results and Discussion

### Two-step correction is vital to avoid large bias in ${\hat{N}}_{e}$ with Pool-seq data

**Figure 2:**
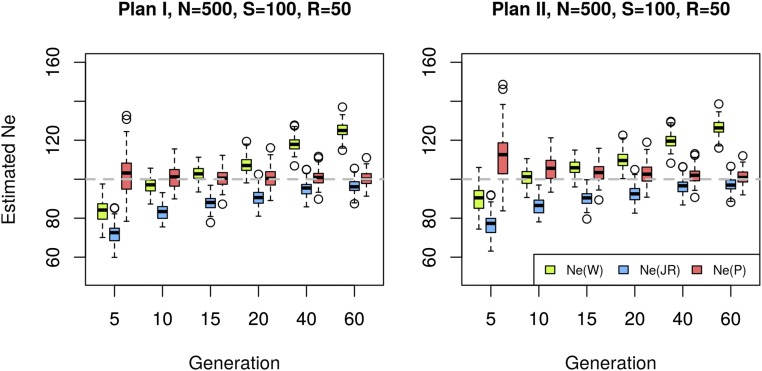
Effective population size estimated with different methods. Sixty generations of Wright–Fisher neutral evolution with $N_{e}=100$ diploid individuals were simulated for *n* = 2000 unlinked loci (SNPs). Prior to sampling, the population was increased to a census size of N=500 individuals at each generation. At the starting population and at each indicated time point a sample was taken to create a pool of S=100 individuals. The pool was sequenced to an average coverage of R=50 and $N_{e}$ was estimated on the resulting data set by separately contrasting allele frequencies at generation 0 to each of the evolved generations denoted on the *x*-axis, using $N_{e}\left(P\right),$ $N_{e}\left(W\right)$ (Waples 1989), and $N_{e}\left(JR\right)$ (Jorde and Ryman 2007). Each box represents results from 100 simulations with identical parameters. The dashed gray line shows the true value of $N_{e}.$ Data are simulated under plan I assumptions and the results of plan I and II estimators are shown in the left and right panels, respectively.

Methods that do not correct for the additional sampling step caused by pooling can lead to substantial bias in ${\hat{N}}_{e}$ as illustrated in Figure 2. Using simulated data, we compare our proposed estimator $N_{e}\left(P\right)$ to two commonly used estimators $N_{e}\left(W\right)$ (Waples 1989) and $N_{e}\left(JR\right)$ (Jorde and Ryman 2007) that provide highly accurate estimates when only a single sampling event is simulated (Figure S3). Figure 2 shows that the additional correction substantially decreases the bias for almost all scenarios (see also Figure S4, Figure S5, and Figure S6). Under plan I, $N_{e}\left(P\right)$ is nearly unbiased. The plan II version of the estimator has a slight upward bias when applied on data simulated under plan I, if the samples are taken at very close time points.

As an alternative approach, we also estimated $N_{e}$ separately for each locus, using ${F^{\prime }}_{c}$ in Equations 9 and 11. We then calculated the effective population size across the *n* loci as the harmonic mean over the single-locus ${\hat{N}}_{e}$ estimates (${\hat{N}}_{e}^{*}\left(P\right)$) (Peel *et al.* 2013). In our simulations, the harmonic mean estimator shows an accuracy similar to that of the original ${\hat{N}}_{e}\left(P\right)$ (Figure S7). However, for *t* lying in the midrange of the simulated interval (t= 15–40), ${\hat{N}}_{e}^{*}\left(P\right)$ is slightly more biased under plan I.

Because of the additional sampling variance, both $N_{e}\left(W\right)$ and $N_{e}\left(JR\right)$ have a downward bias in particular for small *t*. Furthermore, $N_{e}\left(W\right)$ is upwardly biased for larger values of *t*, probably reflecting that alleles closer to fixation or loss are contributing less to the variance (Waples 1989). The drift variance accumulates with an increasing number of generations, while the sampling variance stays constant, making the initial bias of $N_{e}\left(JR\right)$ less pronounced for larger *t*. When samples are collected only a few generations apart, the variance of $N_{e}\left(P\right)$ estimators tends to be larger than that of $N_{e}\left(W\right)$ and $N_{e}\left(JR\right)$ under both plans.

Plan I and II estimators differ by a factor resulting from the covariance between the sample frequencies at generations 0 and *t* (Equation 10), which is inversely proportional to the census population size. Consequently, the difference between plans I and II becomes smaller for increasing *N*. Waples (1989) investigated how the ratio between census and effective population size ($r=N/N_{e}$) affects the accuracy of the estimators and concluded that the ratio of r≥2 is sufficient to reach similar estimates for both sampling schemes. We tested the performance of $N_{e}\left(P\right)$ on simulated data with $N_{e}=100$ and $N:N_{e}$ ratios of r=1,2,5 with different coverages and pool sizes (Figure S4, Figure S5, and Figure S6). When $N=N_{e},$ the $N_{e}\left(P\right)$ plan I method achieves highly accurate estimates for all time points in contrast to the other methods (Figure S4). If, however, the $N_{e}\left(P\right)$ plan II estimator is applied to data simulated under plan I, we observe an upward bias for small *t*, which improves with an increasing number of generations. This pattern is not unexpected since the missing covariance term becomes less influential in view of the increasing drift variance after several generations. When the entire population is sequenced as a single pool (S=100), the plan II estimators of Waples (1989) and Jorde and Ryman (2007) perform similarly to the $N_{e}\left(P\right)$ plan I estimator because the correction for pooling in $N_{e}\left(P\right)$ cancels out the additional covariance term when S=N, making the term used as *F* approximately identical to that of $N_{e}\left(JR\right).$ This is a general pattern irrespective of *r*.

For r≥2, $N_{e}\left(P\right)$ plan I remains highly accurate (Figure S5 and Figure S6). Furthermore, when increasing the census size under a constant $N_{e}$ (equivalent to increasing *r*), the covariance between sample allele frequencies decreases, making the difference between plans I and II almost negligible (Waples 1989). The sampling variance becomes proportionally smaller compared to the drift variance with an increasing number of generations between the samples. This improves our ability to accurately estimate $N_{e}.$

Correcting for the additional variance inherent to Pool-seq leads to an improved performance of $N_{e}\left(P\right)$ compared to the standard methods for both plans. In general, with Pool-seq data the extent of the bias of the $N_{e}\left(W\right)$ and $N_{e}\left(JR\right)$ estimates depends on the ratio between *N* and *S*, smaller sample sizes (*S*) leading to a larger bias. As we accounted for the sequencing step with these estimators (*Estimating $N_{e}$* *on simulated data*), decreasing the coverage at a given pool size does not change the bias much but rather increases the variance of the estimators.

In most of the experimental studies the investigator has control over the census population size; thus requiring the knowledge of *N* for $N_{e}\left(P\right)$ plan I does not restrict the analysis. We illustrate the performance of $N_{e}\left(P\right)$ plan I only when $N_{e}=N$ in the main text, but according to Figure S5 and Figure S6, $N_{e}\left(P\right)$ plan I is also highly accurate when r≥2.

**Figure 3:**
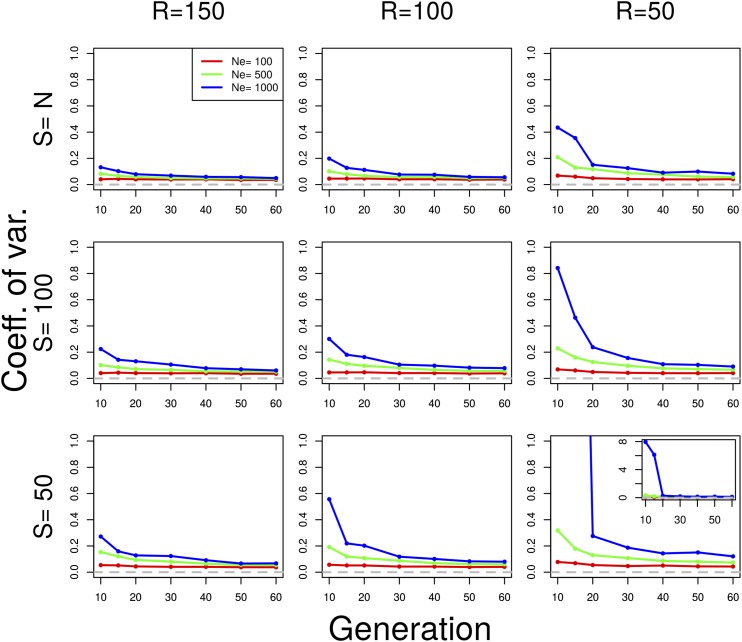
Coefficient of variation of $N_{e}\left(P\right)$ under plan I for various parameter values. Neutral Wright–Fisher simulations were performed with various combinations of the parameters: effective population size ($N_{e}=100,\;500,\;1000$ diploid individuals), pool size (S=100,50), and coverage (R=150, 100, 50). $N_{e}$ was estimated with $N_{e}\left(P\right)$ under plan I, using n=2000 SNPs. S=N indicates scenarios when the whole population is sequenced as a single pool. For all simulations, we assumed $N=N_{e}.$ Each value is calculated over 100 simulations. When the coefficient of variation exceeds one, the inset shows the actual value.

We show the coefficient of variation (CV) of the $N_{e}\left(P\right)$ plan I estimator in Figure 3. The CV is defined as the ratio between the standard deviation and the mean ($\mathrm{CV}=\hat{\sigma }/\hat{\mu },$ where both $\hat{\sigma }$ and $\hat{\mu }$ are estimated from the sample). It measures the relative dispersion of the distribution of the estimated values. $N_{e}\left(P\right)$ estimators are highly precise in nearly all cases, except when the drift variance is negligible compared to the sampling variance (Figure 3; see also Figure S9 and Figure S11 where $N_{e}=1000,\;t<30,\;S\leq 100,$ and R=50). The bias is coming from a few outlier estimates, but the median shows more robust results (Figure S13). For plan II estimators, the behavior of the method is similar (Figure S8, Figure S10, Figure S12, and Figure S14). Note that the simulations underlying Figure S8, Figure S10, Figure S12, and Figure S14 have been done under plan I.

### Increasing the number of SNPs reduces the variance of $N_{e}\left(P\right)$

**Figure 4:**
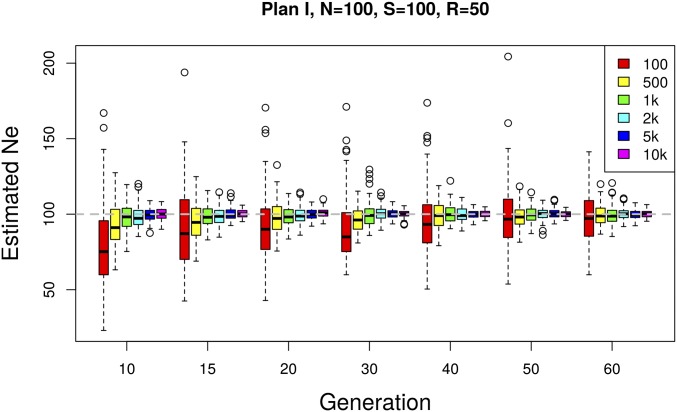
Effect of the number of SNPs used for estimating $N_{e}.$ The effective population size is estimated using $N_{e}\left(P\right)$ plan I on simulated data with $N_{e}=N=100.$ A total number of S=100 individuals are pooled and sequenced at a mean coverage of R=50. Based on 100 simulation runs, $N_{e}$ is estimated using different numbers of SNPs at multiple time points.

We test how the number of loci (*n*) used to infer $N_{e}$ affects the accuracy and the precision of the estimates by gradually increasing the number of independent SNPs from 100 to 10,000 (Figure 4). We observe a larger variance and a slight downward bias for a small number of SNPs (100 SNPs). Both the bias and the variance become smaller with a larger number of SNPs. Some further improvement is obtained when >10,000 SNPs are used (not shown), but the benefit of additional independent SNPs levels off. We conclude that n=2000 SNPs usually provide sufficient precision and accuracy. However, when linkage disequilibrium is present in a genome-wide data set, the number of truly independent SNPs per window is reduced and a larger number of loci is recommended.

### A skewed starting allele frequency distribution only moderately increases the variance of $N_{e}\left(P\right)$

**Figure 5:**
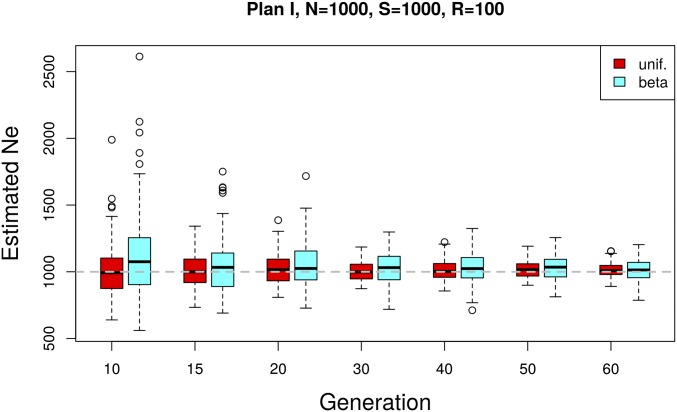
Influence of the starting allele frequency distribution on $N_{e}\left(P\right)$ under plan I. A comparison between uniform and Beta(0.2, 0.2)-distributed (neutral) starting allele frequencies is shown. The simulation parameters match those of the genome-wide simulations in Figure 6.

In natural populations, the neutral site frequency spectrum is skewed toward allele frequencies close to the boundaries. $N_{e}\left(P\right)$ uses a weighting scheme that is not very sensitive to this skew (see also Jorde and Ryman 2007). This makes it robust with respect to the shape of the starting allele frequency distribution. We illustrate this with a simulated data set having a starting allele frequency distribution that is skewed toward low- and high-frequency variants (Beta(0.2, 0.2)) as expected under neutrality. The estimates of $N_{e}$ from such data sets are compared to simulated data with matching parameters but uniform starting allele frequency distribution (Figure 5). We observe a very slight upward bias with neutral starting allele frequencies compared to uniform and a moderate increase in the variance given t≥15. With an increasing number of generations, the difference becomes negligible.

### The presence of linkage disequilibrium does not have a large effect on the precision of $N_{e}\left(P\right)$

**Figure 6:**
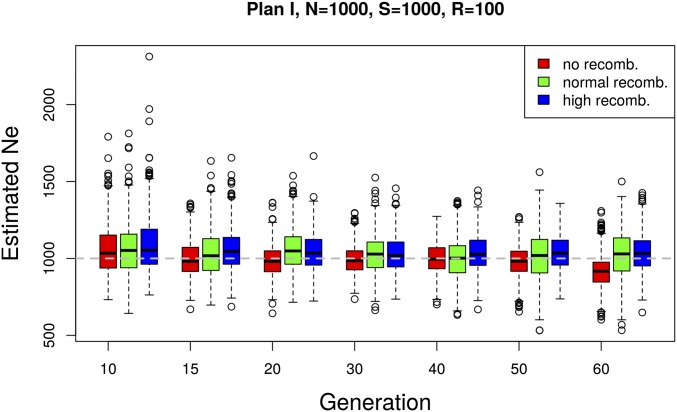
Effect of linkage disequilibrium on ${\hat{N}}_{e}.$ The effect of linkage disequilibrium on our estimator was evaluated based on a whole-genome forward simulation with recombination using the software MimicrEE (Kofler and Schlötterer 2014). Three sets of simulations were performed with different rates of recombination: high, normal, and no recombination. For each parameter setup, a genome-wide simulation is replicated 10 times. The effective population size was estimated with $N_{e}\left(P\right)$ (plan I) in nonoverlapping windows of *n* = 10,000 SNPs for each replicate. The box plots show the distribution of $N_{e}$ estimates across replicates and windows.

We investigated the sensitivity of our estimator to linkage disequilibrium between loci, using genome-wide neutral simulations with recombination (Kofler and Schlötterer 2014). We simulated data with three different rates of recombination: high, normal, and no recombination. For the first case, the recombination rate is set to mimic the behavior of almost independent SNPs. In the normal recombination rate scenario, we use *D. melanogaster* recombination rates (Fiston-Lavier *et al.* 2010). The effective population size was estimated in nonoverlapping windows with a fixed number of n=10,000 SNPs (Figure 6). Different levels of linkage disequilibrium affect the number of independent loci per window. Nevertheless, we observe only a slight increase in the precision of the $N_{e}$ estimates with increasing recombination rate (Figure 6).

### Heterogeneous ${\hat{N}}_{e}$ along the genome in an E&R study with *D. melanogaster*

**Figure 7:**
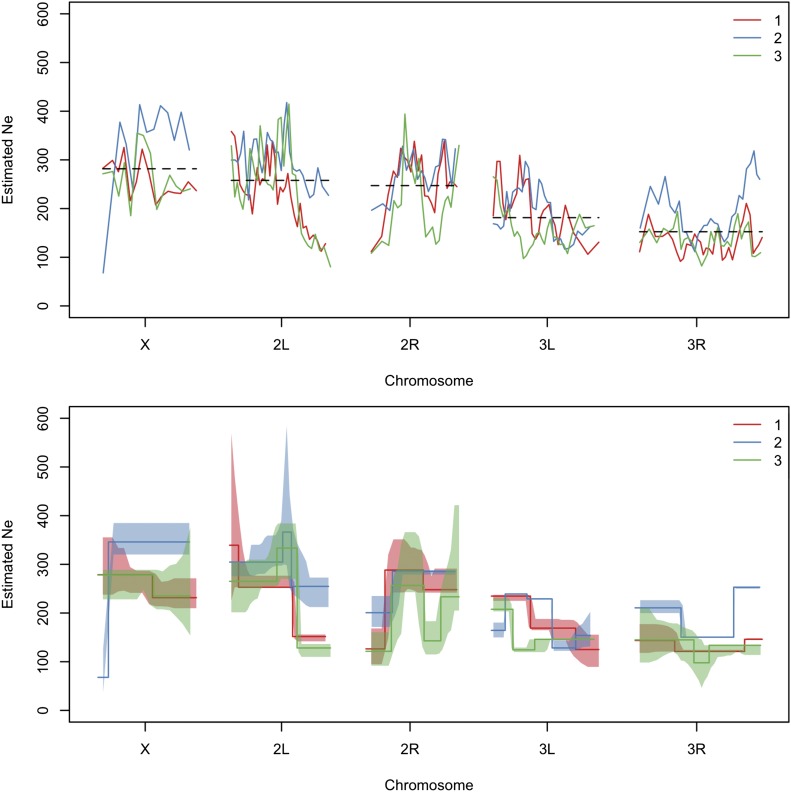
Genome-wide ${\hat{N}}_{e}$ from an E&R study with *D. melanogaster*. $N_{e}$ is estimated based on the allele frequency changes between founder and evolved populations at generation 59 (Franssen *et al.* 2015). In the top panel, we show genome-wide estimates calculated with $N_{e}\left(P\right)$ (plan I), using N=1000 as census size and S=500 as pool size (Orozco-terWengel *et al.* 2012) and nonoverlapping windows of 10,000 SNPs. Chromosome-wide mean estimates across replicates are shown by the dashed lines and also calculated separately for each replicate in Table 1. DNA stretches with significantly different ${\hat{N}}_{e}$ are determined using the *stepR* software package (Frick *et al.* 2014) (bottom panel). Lower and upper 1−α confidence bands are shown as shaded areas. *α* controls the error, *i.e.*, the probability for overestimating the number of change points, and is calculated automatically as described in Frick *et al.* (2014). The colors indicate different biological replicates.

We estimated $N_{e}$ in a recent E&R study with *D. melanogaster* (Orozco-terWengel *et al.* 2012; Franssen *et al.* 2015). In this experiment replicate populations of 1000 individuals were subjected to a fluctuating hot environment for 59 generations. Allele frequency estimates were obtained for founder and evolved populations, using Pool-seq. $N_{e}$ was estimated based on the allele frequency changes between founder and latest evolved populations in nonoverlapping windows containing 10,000 SNPs, using $N_{e}\left(P\right)$ under plan I. To determine the number of DNA stretches with different ${\hat{N}}_{e}$ along the genome, we use a segmentation algorithm provided in the software tool by Frick *et al.* (2014). This method requires homogeneity of variances. Since the variance of estimates increases with $N_{e},$ the estimates were log-transformed before applying the partitioning procedure. The obtained step function was back-transformed to the original scale and is shown for three biological replicates (Figure 7).

The mean and the median estimates for each chromosome arm as well as across the genome are stable across replicates (see Table 1 and Table S1). As experimental evolution studies often aim to find signals that are consistent across replicates, this can be an important check of the experimental setup. On the other hand, we see differences between chromosome arms. For example, the mean is clearly lower for 3R, emphasizing the added value of spatial analysis compared to genome-wide estimates.

**Table 1:** Genome-wide mean ${\hat{N}}_{e}$ from an E&R study with *D. melanogaster*

	Mean					
Replicate	X	2L	2R	3L	3R	Genome-wide
R1	257.9675	231.6854	257.0828	193.4339	131.7072	199.4463
R2	328.8878	297.9832	274.8529	193.3237	194.9571	239.3618
R3	263.4829	246.5448	211.8995	157.6411	133.9459	187.1573

The effective population size is estimated with $N_{e}\left(P\right)$ plan I in windows of 10,000 SNPs (Figure 7). The mean estimates across windows are shown for the major chromosome arms. The genome-wide mean is taken over the autosomes, excluding chromosome 4.

In *D. melanogaster* ${\hat{N}}_{e}$ ranges between ∼100 and 400. Around the centromere of chromosome 2, the estimated $N_{e}$ decreases by two-thirds in replicates 1 and 3, which is in agreement with the expectation of low diversity and, as a consequence, low $N_{e}$ in regions with reduced recombination (Begun and Aquadro 1992; Presgraves 2005; Haddrill *et al.* 2007; Campos *et al.* 2012). Furthermore, ${\hat{N}}_{e}$ is low on the entire chromosome arm 3R and also on parts of 3L. Overall, these patterns can be attributed to strong LD, caused either by low recombination rates around the centromeres (Chan *et al.* 2012) or by segregating inversions (Kapun *et al.* 2014) in combination with selection potentially on rare variants. The reduction in ${\hat{N}}_{e}$ is also well captured by the segmentation algorithm (Figure 7), which shows a similar pattern when applied on simulated data with selection (Figure S15). These results are consistent with those of Tobler *et al.* (2014), who observed a massive amount of outlier SNPs around the centromere of chromosome 2 and on 3R. Interestingly, certain regions of the genome show extensive differences in ${\hat{N}}_{e}$ between the replicates, which might be reflecting different selection histories or differences in demography, such as replicate-specific bottlenecks.

${\hat{N}}_{e}$ may also vary as a result of differences in the modes of transmission of different components in the genome. For example, on the X chromosome, $N_{e}$ is equal to three-quarters of the autosomal population size (Vicoso and Charlesworth 2006, 2009). Interestingly, our estimates in the E&R experiment do not reflect this expectation of reduced effective population size. Instead, we estimate $N_{e}$ to be as high as ${\hat{N}}_{e}$ on the autosomes. Unequal sex ratio between males and females can be a source of such a pattern (Charlesworth 2009); however, unbalanced sex ratio has not been reported in this experiment. Another possible explanation for increased ${\hat{N}}_{e}$ on the X can be the presence of background selection as suggested by Charlesworth (2012b). He argues that because of the lack of recombination in male *Drosophila*, the effect of background selection is more effective on the autosomes than on the X chromosome. Orozco-terWengel *et al.* (2012) reported differences in the number of putatively selected sites between the X and autosomes. They found that candidate SNPs were underrepresented on the X. Their selection scan identifies signatures of deviation from neutral expectation, which is also reflected in the reduction in ${\hat{N}}_{e}$ on the autosomes, indicating higher selection pressure.

### Recommendations for genome-wide data sets

Most of the methods proposed previously are not designed for genome-wide high-density SNP data sets. However, the method of Jorde and Ryman (2007) was successfully used for genome-wide data by Foll *et al.* (2014). Reed *et al.* (2014) also used a similar approach to estimate $N_{e}$ for whole-genome data, using sliding windows. We estimated $N_{e}$ in windows with a fixed number of SNPs. Using windows of fixed lengths in base pairs would affect the variance of the estimator (Figure 4) but does not distort the mean. All these approaches, however, do not account for the ruggedness of the recombination landscape and can lead to windows with different levels of linkage disequilibrium in them. To overcome this problem it would be possible to define windows based on recombination distance. Unfortunately, the lack of haplotype information in the Pool-seq data makes it difficult to infer linkage disequilibrium. One way to infer linkage information from pooled sequence data is provided by the software LDx (Feder *et al.* 2012). For model organisms such as *Drosophila*, readily available recombination maps can also be used as a proxy (Przeworski *et al.* 2001; Kulathinal *et al.* 2008; Fiston-Lavier *et al.* 2010). If only a single genome-wide $N_{e}$ estimate is required, one can alternatively use a set of randomly distributed SNPs over the genome to obtain ${\hat{N}}_{e}.$

Temporal methods make a number of assumptions, which, if violated, can lead to biased $N_{e}$ estimates. For example, in our simulations, we considered only effective population sizes that are constant over time. Fluctuating $N_{e}$ is a frequent phenomenon in natural populations and can be an important component of an experimental design. For example, in repeatedly bottlenecked populations, the smallest population size dominates ${\hat{N}}_{e}$ (Luikart *et al.* 1999; Charlesworth 2009). But even in strictly controlled populations the experimental regime can induce changes in $N_{e}.$ When the population changes in size, the estimated $N_{e}$ is generally interpreted as the harmonic mean of the effective population sizes over the generations (Wright 1938; Nei and Tajima 1981; Waples 1989). However, if time series allele frequency data are available, such changes can be detected by estimating $N_{e}$ from pairwise comparisons between consecutive time points.

All evolutionary forces (selection, demography, etc.) that lead to deviations from the neutral expectation will also affect our estimate. Nevertheless, systematic forces that result in locally different values of ${\hat{N}}_{e}$ can be detected with a sliding-window approach, as illustrated with simulations under selection (Figure S15). The *D. melanogaster* data set also illustrates this point; *i.e.*, the hypothesized regions under selection coincide with regions of reduced $N_{e}$ (Orozco-terWengel *et al.* 2012; Tobler *et al.* 2014; Franssen *et al.* 2015). For this to be detected, however, most of the allele frequency change has to occur over the sampled time span.

In the E&R study with *D. melanogaster*, shown above, the criterion of nonoverlapping generations, assumed by temporal methods, is met (see Orozco-terWengel *et al.* 2012 for details on experimental design). However, for samples from an age-structured population, the resulting ${\hat{N}}_{e}$ can be biased (Waples and Yokota 2007). In these cases, as suggested by Waples and Yokota (2007), larger spacing between samples maximizes the drift signal compared to sampling biases associated with age structure.

### Using a small number of generations can lead to outlier estimates

In general, $N_{e}\left(P\right)$ has a lower bias but larger variance, especially when *t* is small. As pointed out by Jorde and Ryman (2007) our weighting scheme leads to an increased variance but a smaller bias compared to other schemes. We observe outlier estimates among replicates at early generations (generation 5, Figure 2, Figure S4, Figure S5, and Figure S6) for $N_{e}\left(P\right).$ When the sampling variance is large compared to the drift variance ($N_{e}=1000,$ S≤100, and R=50, Figure S11 and Figure S12), the deviation of the outlier estimates from the true $N_{e}$ is particularly large. For a few cases, we even observe large negative estimates. Negative estimates, in general, can be interpreted as $N_{e}$ being infinity, that is, no evidence of genetic drift (Peel *et al.* 2013). In our simulations this is plausible when $N_{e}$ is large and *t* is small, such that drift has not had a large effect on the population allele frequencies yet. Note that the harmonic mean estimator (${\hat{N}}_{e}^{*}\left(P\right)$) has smaller variance for large $N_{e}$ (Figure S16). This estimator, however, is less accurate than $N_{e}\left(P\right)$ for small $N_{e}$ as shown in Figure S17.

To eliminate potential outliers and an inflated variance we recommend increasing the signal-to-noise ratio by pooling a sufficient number of individuals. Using later generations or increasing the number of SNPs in the analysis also helps to avoid outlier estimates. When none of these strategies can be applied, we suggest using the genome-wide median $N_{e}\left(P\right)$ estimates or the harmonic mean estimator, as these are more robust to extreme outliers.

### Conclusions

Effective population size is an important parameter for describing evolutionary dynamics, making its accurate estimation essential for population genetic studies. Several methods have been designed to estimate $N_{e},$ and their performance was comprehensively evaluated on simulated as well as real data (Barker 2011; Serbezov *et al.* 2012; Baalsrud *et al.* 2014; Holleley *et al.* 2014; Gilbert and Whitlock 2015). These studies mainly focused on genetic data collected from natural populations, which usually differ from experimental studies in terms of the census population size and sampling scheme. We designed a method that accurately infers the effective population size in genome-wide data from experimental populations sequenced in pools. Our approach improves temporal methods by explicitly correcting for two stages of sampling introduced by pooling and sequencing. Our results on simulated data confirm that methods that fail to properly account for the two stages of sampling inherent to Pool-seq can lead to severely biased $N_{e}$ estimates.

Pool-seq data are often considered to be overdispersed, *i.e.*, displaying more variability than is predicted by the binomial sampling model (Yang *et al.* 2012). However, Zhu *et al.* (2012) and Futschik and Schlötterer (2010) validated that the error in allele frequency estimates is reasonably well approximated by binomial sampling given that a large enough number of individuals are pooled. Nevertheless, if overdispersion is present in the data, that will lead to additional variance, which is not modeled in our framework and will result in a downward bias of the estimated $N_{e}.$ If the level of overdispersion can be inferred for the data (see, *e.g.*, Gautier *et al.* 2013; Illingworth 2015), it is possible to introduce a parameter that accounts for the additional between-pool variation (see File S1, Equation S8).

We also illustrate the applicability of our method for estimating $N_{e}$ from experimental data of *D. melanogaster* and show that in combination with a recursive partitioning method we can infer patterns of local variation in $N_{e}$ along the genome. Additionally, it is possible to calculate confidence intervals based on the $\chi^{2}$ distribution (Waples 1989) or alternatively apply a nonparametric bootstrap approach.

### Software availability

Our proposed estimators along with standard methods from the literature are implemented within the R package *Nest*. The package is currently available at https://github.com/ThomasTaus/Nest.

## 
